# Common microbial signatures in blood and their amplification in clinical disorders

**DOI:** 10.1080/29933935.2025.2473450

**Published:** 2025-03-19

**Authors:** Gwoncheol Park, Suji Oh, Minjeong Kim, Yunsun Jeong, Gyungcheon Kim

**Affiliations:** a Department of Health, Nutrition, and Food Sciences, College of Education, Health, and Human Sciences, Florida State University, Tallahassee, Florida, USA; b Department of Food Science & Biotechnology, College of Life Science, Sejong University, Seoul, Republic of Korea

**Keywords:** Blood, blood microbiome, microbial signature, cell-free DNA, meta-analysis

## Abstract

Blood microbiome research has emerged as a significant area of study, exploring microbial signatures within the bloodstream and their potential implications for various clinical disorders. This study aimed to identify common microbial signatures in blood across cohorts and investigate how these signatures are altered in clinical conditions. We conducted a meta-analysis of 15 publicly available studies utilizing amplicon sequencing, including 687 control and 651 case individuals with various disorders from diverse geographic locations to compare their blood microbiome profiles. The results revealed that most microbes detected in the blood originated from the gut, oral cavity, and skin, with several genera such as *Corynebacterium*, *Streptococcus*, and *Lactobacillus* consistently identified across studies. Furthermore, we observed a significant increase in microbial diversity and abundance in individuals with clinical disorders compared to the control group. Notably, microbial genera originating from the gut and oral cavity, including *Acinetobacter*, *Prevotella*, and *Clostridium sensu stricto-1*, were more prevalent in disease cohorts, suggesting a potential link between the translocation of microbial signatures and disease pathology. The study underscores the importance of considering microbial signatures as potential biomarkers in clinical settings and calls for further investigation into the role of circulating microbial DNA in immune response and disease progression.

## Introduction

Traditionally, blood has been considered sterile in healthy individuals because various bacteriostatic and bactericidal components—such as antibodies, complement proteins, and white blood cells—contribute to the immune response and inhibit microbial infection.^33,67^ However, recent studies have demonstrated that microorganisms can be present in the blood, depending on the host’s health status, and may interact with the immune response.^12,16,100^ Blood consists of approximately 45% erythrocytes (red blood cells), less than 1% leukocytes and platelets (collectively known as the buffy coat), and about 55% plasma. Notably, most microbial DNA in blood resides in the buffy coat, accounting for approximately 93.74% of the total.^79,83^ This microbial DNA is generally thought to translocate from microbiota-rich areas, such as the gut and oral. The translocation of gut microbiota into the bloodstream has been shown to increase when inflammation or dysbiosis compromises intestinal permeability, allowing pathogenic bacteria to enter systemic circulation and promoting various bloodborne diseases.^24,32,42,86,87,88,90^ Inflammatory skin disorders can also weaken the skin barrier, facilitating microbial translocation into the bloodstream and increasing the risk of systemic inflammation and disease.^57,65,81,112^ In addition to live bacteria, microbial cell-free DNA (cfDNA) can also activate the immune system. Bacterial DNA contains Cytosine-phosphate-Guanine (CpG) motifs, which act as pathogen-associated molecular patterns (PAMPs).^61,128^ These motifs are recognized by toll-like receptor 9 (TLR9), a pattern recognition receptor in immune cells, triggering a cascade of immune responses. Thus, the microbial DNA load in the blood can reflect host health and chronic inflammation status.

The analytical methods for studying the blood microbiome are continually evolving. Recent advancements in high-throughput next-generation sequencing (NGS), including amplicon and shotgun metagenome sequencing, have significantly improved our understanding of blood microbiota, enabling deeper exploration of its composition and role.^12^ However, metagenomic sequencing in blood analysis presents several challenges, such as the high prevalence of host-derived DNA, which can obscure microbial signals, and difficulties in detecting low-abundance microorganisms.^102^ Host-derived DNA and contamination during sample handling often outweigh microbial signatures in blood, leading to an increase in off-target reads and reducing the likelihood of detecting low-biomass bacterial signatures.^28^ Specifically, high proportions of host DNA significantly decrease the sensitivity for detecting low-abundance microbes in metagenomic shotgun sequencing by reducing the probability of microbial DNA being sequenced. Similarly, excessive host DNA poses challenges in amplicon sequencing due to off-target amplification. Although amplicon sequencing targets specific regions of microbial genomes, off-target amplification of host DNA is frequently observed in samples with a high host-to-microbial DNA ratio, such as biopsy samples.^22^ The detection rate of off-target amplification is strongly influenced by primer selection, with primers targeting the V4 region of bacterial 16S rRNA yielding up to 98% off-target reads.^22^ Moreover, amplification not only targets microbial genomes but can also amplify exogenous contaminants, misrepresenting extremely low-biomass samples as having high bacterial loads.^121^ For example, a study on the brain microbiome revealed that 89% of reads were exogenous DNA contamination or off-target host DNA amplicons.^7^ Additionally, DNA sequencing can detect both live bacterial DNA and cfDNA, requiring additional validation to differentiate between viable bacteria and fragmented bacterial DNA.

Indeed, previous studies attempting to capture bacterial signatures in blood and their alterations in disease have reported issues with contamination. For instance, a study comparing the blood microbiome of healthy individuals with asthma patients detected microbes primarily from oral and skin sources, such as *Staphylococcus* spp.^122^ Similarly, research on viable bacteria in freshly drawn blood samples found viable bacteria in 62% of healthy individuals, with *Propionibacterium acnes* and *Staphylococcus epidermidis*—common skin bacteria – identified as the predominant species.^20^ Both studies emphasized that the detection of microorganisms in blood samples may result from contamination during handling. Additionally, contamination from laboratory equipment during experimental procedures can lead to inaccurate reports of the blood microbiome.^36,96,97,98,99,101^ These findings highlight the importance of strict protocols during sample collection and rigorous control measures to accurately assess the blood microbiome. While aseptic techniques can significantly reduce contamination risks, completely eliminating contamination remains challenging, underscoring the need for advanced bioinformatics strategies to filter potential contaminants. A recent large-cohort study on the common blood microbiome applied extensive strategies to remove contaminants from shotgun metagenome sequencing data, revealing that most sequences were eliminated during quality control. The remaining data suggested that human blood does not maintain a stable community of resident microbes.^109^ However, since this study utilized shotgun metagenome sequencing – known to have limitations for samples with high host-derived DNA and low microbial biomass, like blood – comprehensive studies using more targeted sequencing strategies are still needed. Meanwhile, studies on the blood microbiome using amplicon sequencing have reported considerable diversity in microbial communities, although the major microbes composing the blood microbiome vary between studies.^82,91,126,127^ Given the limitations of amplicon sequencing, extensive quality control strategies, including host-derived DNA filtration and decontamination with negative controls, need to be integrated into these studies. However, the studies did not consistently apply all of these strategies, which may result in some variability in the blood microbiome profiles reported. This highlights the need for further refinement in methodological approaches to enhance the accuracy of microbial community characterization in blood samples. Such approaches, which focus specifically on microbial DNA, may offer advantages and yield clearer insights into the blood microbiome, but these studies have yet to be conducted.

Driven by this research gap, we conducted a meta-analysis of 15 publicly available blood microbiome datasets from diverse geographic locations, using amplicon metagenome sequencing to characterize the common blood microbiome and assess how it changes in patients with clinical disorders. To ensure more accurate characterization, we employed multiple strategies to eliminate human-derived and potential environmental contaminants, and all analyses were performed on decontaminated data. Based on our findings, we discussed the sequencing techniques used in blood microbiome analysis, the common microbial signatures shared across individuals, and how these signatures are altered in clinical disorders.

## Materials and methods

### Study selection

**Figure 1.**
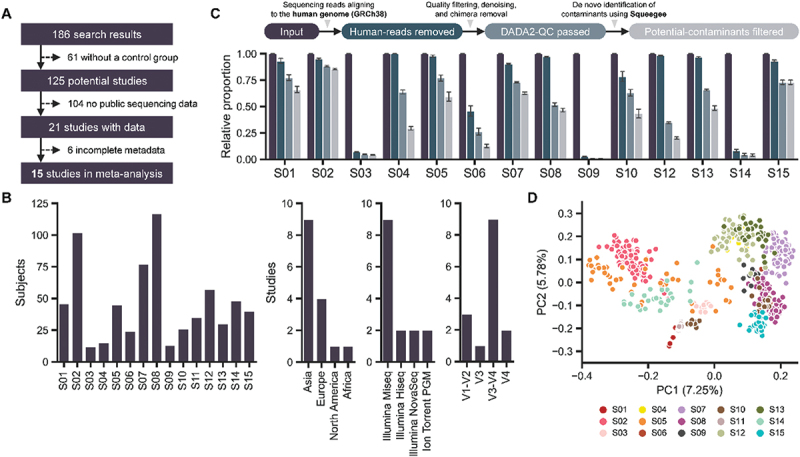
Study characteristics. (A) Flow diagram of study inclusion (B) number of subjects per study and study profiles, including the continent of study, sequencing platform, and amplified region. (C) Relative proportion of sequences at each quality control step. Human reads were removed by alignment to the human genome (GRCh38), and filtered reads were processed through DADA2 quality control, followed by the removal of potential contaminants identified with Squeegee. (D) PCoA plot displaying sample distribution based on unweighted UniFrac distances. Data are presented as mean ± SE.

Case-control 16S amplicon sequencing studies were identified through keyword searches in PubMed and the NCBI Sequence Read Archive (SRA) in August 2024. Search terms include: (“blood” OR “serum” OR “plasma” OR “circulation”) AND (“microbiome” OR “microbial” OR “bacterial” OR “cfDNA”) AND (“homo sapiens” OR “human” OR “patient” OR “participant”) AND (“16S” OR “amplicon” OR “metabarcoding”). We included only original research, such as clinical trials and observational studies, without restricting the time frame in which the studies were conducted. The search initially returned 186 potential studies. Only those with publicly available 16S rRNA sequencing data in FASTQ format, along with metadata clearly indicating case or control status for each sample, were considered. Additionally, studies requiring further ethics committee approval or special authorization for data access were excluded. To ensure robustness, only human cohort studies were included, and those with fewer than ten case or control subjects were excluded. Ultimately, 15 studies were selected for meta-analysis (Figure 1A). The data were downloaded from the NCBI SRA and Open Science Framework (OSF). The sequencing data uploaded to NCBI SRA were downloaded using the Python package *q2-fondue*,^129^ incorporated with the QIIME 2 platform, and directly utilized for microbiome profiling. Some studies focused on characterizing the microbiome of different body sites in healthy individuals without a disease cohort and were included in the profiling of healthy individuals, though excluded from case-control comparisons. For longitudinal studies involving specific treatments, only baseline samples collected before any interventions were included in the analysis.

### Data pre-processing and quality control

Sequencing reads were aligned to the reference index built with the latest version of the human reference genome (GRCh38.p14) using Bowtie2^63^ and identified human reads were removed. Sequencing data from various platforms were processed using the DADA2 plugin embedded in QIIME2 (version 2024.5)^9^ platform for denoising paired-end, single-end, and pyrosequencing reads, with default settings (max-ee-f/r = 2, trunc-q = 2). Reads were filtered based on quality scores, denoised, and chimera sequences were removed. Trim lengths during DADA2 processing were adjusted according to the sequencing platform’s read length and the amplified hypervariable region. For paired-end reads, if poor merging resulted in over 50% of the reads failing to merge, only forward reads were used. Amplicon sequence variant (ASV) tables and representative sequences from each study were merged for downstream analysis, with samples containing fewer than 100 reads excluded. All identified ASVs were aligned using MAFFT.^52^ Taxonomy assignment for ASVs was conducted with the sklearn classifier, utilizing a pretrained Naive Bayes taxonomy classifier aligned to the 99% SILVA 138 database.^92^ To eliminate potential microbial contaminants from DNA extraction or environmental sources, *de novo* contamination detection was performed using Squeegee,^72^ and ASVs matching potential contaminant taxa were removed. For microbial diversity assessment, the rarefaction depth was set to 2000 reads. All statistical analyses were conducted on genus-level data, transformed to relative abundance.

### Blood microbiome profiling and statistical analyses

Number of observed ASVs and the Faith’s phylogenetic diversity (PD) were employed as measures of microbial diversity (alpha diversity). For microbial composition (beta diversity) comparisons between studies, unweighted UniFrac distance was used, with the results visualized through principal coordinate analysis (PCoA). To classify the potential body site origin for each microbial genus, a similar approach to a previous study^109^ was employed, with minor adjustments. Briefly, data from 10,866 experiments were obtained from the Disbiome database^47^ (accessed on 18th September 2024), and information on microbes and their sample types was extracted. Sample types were categorized as follows: feces, anal swab, and rectal swab were grouped as gut samples; buccal, dental plaque, mouthwash, oral plaque, oral swab, oral wash, oropharynx swab, saliva, subgingival plaque, supragingival plaque, and tongue scrape were grouped as oral samples. Additionally, skin swabs and foot swabs were categorized as skin samples. Any sample types that could not be clearly matched to body sites were excluded. To more rigorously determine the origin of the genera identified in this study, only sample origins reported in more than five experiments (≥0.1% of total gut experiments, ≥0.5% of total oral experiments, and ≥ 1% of total skin experiments) were considered valid. If a genus was associated with more than two origins, each supported by over five experiments, it was classified as “multi-origin”. Genera with fewer than five experiments or no records were classified as “unidentified.” For comparisons between control and case groups in microbial composition, the ANOVA-Like Differential Expression 2 (ALDEx2)^31^ method was employed, with significance determined by the Benjamini-Hochberg corrected p-value (q-value) from the Wilcoxon rank-sum test. All visualizations were generated using Python (version 3.8.16) packages.

## Results

### Study selection and characteristics

To investigate whether there is a common blood microbiome signature shared among normal individuals, regardless of geographic location and experimental setting, and to assess whether these characteristics can be identified through amplicon sequencing strategies, studies that focus on blood microbiomes with publicly available raw 16S amplicon sequencing data and metadata were included. Only studies with clearly distinguishable control and case samples, consisting of at least ten control individuals, were considered. As a result, out of the 186 studies initially identified, 15 met the criteria and were successfully downloaded and processed for meta-analysis (Figure 1A, Table 1). In total, 687 control individuals from these studies were included, with sample sizes ranging from 12 to 117 subjects. Notably, 80% of the participants (n = 550) were clearly identified as healthy at the time of sample collection. Most samples were collected from Asian countries, including China and South Korea, followed by those from European countries. The Illumina MiSeq was the predominant sequencing platform, with the 16S V3-V4 hypervariable region being the most commonly targeted for amplification, followed by the V1-V2 region (Figure 1B).

**Table 1.** Datasets collected and processed for meta-analysis.

ID	Control	Case	Accession No.	Sequencing platform	Target region	Sample type	Year & Ref.
S01	Control patient cohort (*n* = 46)	Patients with liver cirrhosis (*n* = 58)	OSF: T2ED7	Illumina Miseq	V1–V2	Plasma	(2022)^35^
S02	Healthy control cohort (*n* = 102)	Tobacco smoker (*n* = 20) HIV cohort (*n* = 40) Systemic lupus erythematosus cohort (*n* = 19)	NCBI-SRA: PRJNA1477	Illumina Miseq	V4	Plasma	(2021)^74^
S03	Healthy control cohort (*n* = 12)	Severe acute pancreatitis (SAP)-uninfected cohort (*n* = 17) SAP-infected cohort (*n* = 16) SAP-septic cohort (*n* = 17)	NCBI-SRA: PRJNA8535	Thermo Fisher Scientific Ion Torrent PGM	V3	Whole blood	(2018)^69^
S04	Healthy control cohort (*n* = 15)	Takayasu’s arteritis cohort (*n* = 13) giant cell arteritis cohort (*n* = 9)	NCBI-SRA: PRJNA1427	Illumina Miseq	V3–V4	Serum	(2021)^23^
S05	Healthy control cohort (*n* = 45)	Parkinson’s disease cohort (*n* = 45)	NCBI-SRA: PRJNA1524	Illumina Hiseq	V3–V4	Peripheral blood	(2018)^91^
S06	Healthy control cohort (*n* = 24)	Polycystic ovary syndrome cohort (*n* = 24)	NCBI-SRA: PRJNA0247	Illumina Miseq	V3–V4	Whole blood	(2022)^118^
S07	Healthy control cohort (*n* = 77)	Type-1 diabetes cohort (*n* = 64)	NCBI-SRA: PRJNA5939	Illumina NovaSeq	V3–V4	Whole blood	(2024)^125^
S08	Healthy cohort (*n* = 117)	No case group	NCBI-SRA: PRJNA7093	Illumina Miseq	V3–V4	NA	(2023)^46^
S09	Non-septic control cohort (*n* = 13)	Sepsis cohort (*n* = 13)	NCBI-SRA: PRJNA6752	Illumina Miseq	V3–V4	Whole blood	(2022)^107^
S10	Healthy cohort (*n* = 26)	No case group	NCBI-SRA: PRJNA2891	Illumina NovaSeq	V3–V4	Whole blood	(2024)^126^
S11	Healthy control cohort (*n* = 35)	Febrile cohort (*n* = 213)	NCBI-SRA: PRJNA2334	Illumina Miseq	V1–V2	Plasma	(2024)^66^
S12	Healthy control cohort (*n* = 56)	Major depression episode cohort (*n* = 56)	NCBI-SRA: PRJNA6190	Illumina Miseq	V3–V4	Plasma	(2021)^15^
S13	Cognitively normal control cohort (*n* = 30)	Alzheimer’s disease cohort (*n* = 30) Mild cognitive impairment cohort (*n* = 30)	NCBI-SRA: PRJNA9760	Illumina Miseq	V3–V4	Whole blood	(2019)^70^
S14	Normal control cohort (*n* = 48)	Myalgic encephalomyelitis/chronic fatigue syndrome cohort (*n* = 30)	NCBI-SRA: PRJNA2103	Thermo Fisher Scientific Ion Torrent PGM	V1-V2, V4-V5	Whole blood	(2018)^29^
S15	Healthy control cohort (*n* = 40)	Stable coronary heart disease cohort (*n* = 48) ST-segment elevation myocardial infarction cohort (*n* = 94)	NCBI-SRA: PRJEB4590	Illumina Hiseq	V4	Whole blood	(2018)^127^

Blood is expected to contain low microbial biomass alongside high levels of host-associated DNA, which complicates the accurate tracking of microbial signatures in blood samples and can lead to erroneous interpretations of microbial communities.^36,115^ To minimize these issues, several quality control strategies were implemented. First, off-target amplification of human DNA was addressed by aligning sequences to the human genome, resulting in the removal of approximately 20% of total reads (11,429,927 reads). This was followed by filtering and denoising steps using the bioinformatics tool DADA2, which led to an additional loss of 25% of reads (15,067,601 reads). For low-biomass sample sequencing, negative control samples were included to track contamination from the DNA extraction kit and eliminate erroneous microbial signals from sequencing. After checking for contaminants, several computational approaches were employed for effective decontamination, including filtering out sequences present in negative controls and using bioinformatics programs such as Decontam.^21,51^ However, many studies involving low-biomass samples either do not include negative control samples or fail to upload sequencing data even when it exists. Among the studies included in this meta-analysis, only one provided negative control samples. To address this limitation, Squeegee, a computational method for *de novo* contaminant identification, was employed in the absence of negative controls. This method successfully identified potential microbial contaminants from each study, resulting in the removal of nearly 9% of reads (5,241,071 reads) associated with contaminants. Ultimately, approximately 45% of the initial input reads were retained for microbial community profiling (Figure 1C). The overall microbial structure of the studies, as measured by PCoA analysis, exhibited clustering by individual studies, with studies from the same continent and country tending to cluster together. This indicates that cohorts within the same study share similar microbial profiles and exhibit common microbial compositions within their respective sets (Figure 1D, Figure S1).

### Identification of common blood microbiome profiles across diverse control cohorts

**Figure 2.**
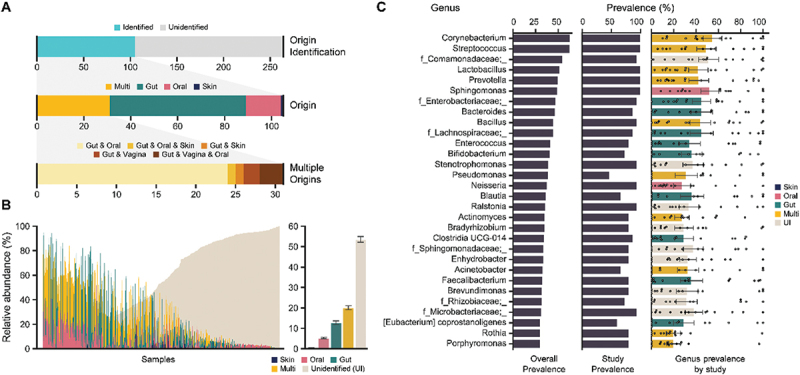
Blood microbiome profiles in control cohort. (A) Expected origin of the blood microbiome. Plots display the number of identified origins based on the Disbiome database (top), the origins of identified taxa (middle), and the types of multiple origins (bottom). (B) Relative proportions of skin-, oral-, gut-, multi-, and unidentified-origin taxa for each sample (left) and the average across all samples (right). (C) Top 30 most abundant genera in the blood microbiome. Overall prevalence across all samples (left), study prevalence (middle), and taxa prevalence by study (right) are shown. Data are presented as mean ± SE.

To identify common microbial signatures in the blood, the microbial composition at the genus level was analyzed across all subjects. For the 264 genera with a prevalence of over 20%, their potential origins were assessed using the Disbiome database, which integrates extensive data on microbial composition changes across various diseases. By collecting information on bacterial presence and where they were found, the potential origins of the genera identified in this study were inferred, resulting in the identification of 105 genera origins. Among these, the majority (58 genera) were exclusively of gut origin, followed by oral-specific genera (15 genera). Thirty-one genera were observed to have multiple potential origins, primarily from both gut and oral sources. A few genera were exclusively from the skin or from both skin and gut, and five genera were identified as originating from both the gut and the vaginal environment (Figure 2A). Based on these data, the overall proportions of microbes from specific origins were calculated. While microbes of unidentified origin represented the largest relative proportion of the blood microbiome, multi-origin, gut-origin, and oral-origin microbes also comprised substantial proportions. Except for nine subjects, all participants harbored at least one multi-origin, gut-origin, or oral-origin microbe. Specifically, gut-origin microbes accounted for an average of 12% of the blood microbiome, with 10% of participants having more than 50% of their blood microbiome composed of gut-origin microbes, while 14% of participants had no detectable gut-origin microbes. Multi-origin microbes comprised 20% on average, with 17% of participants exhibiting over 50% of multi-origin microbes in their blood (Figure 2B).

Although the overall microbial structures varied between studies, several bacterial genera were consistently shared across most studies and subjects. Multi-origin genera such as *Corynebacterium*, *Streptococcus*, and *Lactobacillus* were found in all studies with an overall prevalence exceeding 50%. Several gut-origin bacteria, including an unknown genus in the Enterobacteriaceae family, *Bacteroides*, an unknown genus in the Lachnospiraceae family, *Enterococcus*, and *Bifidobacterium*, were detected in the majority of studies (prevalence ranging from 73% to 93%), with overall prevalence surpassing 40%. Additionally, two bacteria primarily of oral origin, *Sphingomonas* and *Neisseria*, were found in nearly all studies (100% and 93%, respectively) and had substantial prevalence across most subjects (49% and 38%, respectively) (Figure 2C). It remains unclear whether these microbial signals represent microbial cfDNA or the actual presence of live bacteria, as further experiments such as blood cultures are required to make this distinction. Nonetheless, the finding that the blood contains considerable microbial signatures, with several shared across different studies worldwide and present in the majority of individuals, is significant. Given that the bloodstream is not typically a habitat for bacteria and no symbiotic metabolic activities have been reported within the bloodstream, these signatures may be associated with the translocation of cfDNA from organs such as the gut and oral cavity.^38,60^

### Circulating microbial signatures are amplified in various clinical disorders

After identifying common microbial signatures in the blood across various studies, we next explored whether these signatures differed in relation to clinical disorders. Instead of focusing on disease-specific alterations, we examined common and general changes observed across a range of clinical conditions. For this analysis, we included only studies that clearly identified both control (Ct) and case (NCt) groups through metadata, allowing us to utilize 12 studies involving chronic, infectious, autoimmune, inflammatory, neurodegenerative, and cardiovascular diseases.

**Figure 3.**
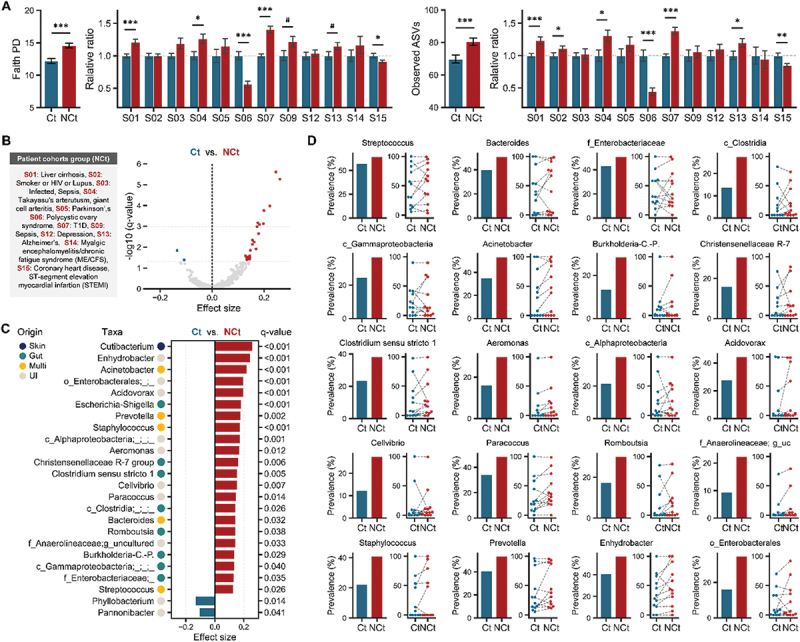
Blood microbiome differences between control and case cohorts with a range of diseases. (A) Bacterial diversity (alpha-diversity) was assessed using Faith’s phylogenetic diversity (PD) and the number of observed ASVs. The left panel shows overall diversity in the ct and NCt groups, while the right panel displays relative diversity normalized to the average of the ct group in each study. Significance was determined using the Kruskal–Wallis test. (B) Volcano plots showing differentially expressed genera (effect size), with significant genera (q < 0.05) highlighted in group-specific colors (blue: Ct; red: NCt) and non-significant genera in grey. (C) Significant differences in blood microbiome composition between groups were analyzed using ALDEx2. (d) Genus prevalence in the ct and NCt groups (left) and prevalence by study (right). Ct and NCt groups from the same study are linked by dotted lines. Only genera with study prevalence greater than 50% among the significantly abundant genera between ct and NCt are shown. #*p* < 0.1; *p < 0.05; **p < 0.01, ***p < 0.001. Ct: control group; NCt: case group. Data are presented as mean ± SE.

Notably, there was a significant increase in microbial diversity (alpha-diversity) in the NCt group compared to the Ct group. This increase was evident not only in species richness (observed ASVs; p < 0.001) but also in the presence of genetically more diverse taxa (Faith’s phylogenetic diversity (PD), p < 0.001). When examining each study, the majority showed substantial increases or a trend toward increased diversity, except for a few studies focused on chronic diseases (Figure 3A). In addition to changes in microbial diversity, significant enrichment of various genera was primarily observed in the NCt group (Figure 3B,C). Specifically, 22 genera, mostly originating from the gut or both the gut and oral, were significantly enriched in the NCt group, while only two genera were more abundant in the Ct group (Wilcoxon rank-sum test, Benjamini-Hochberg corrected p-value (q-value) <0.05). Two of these genera, *Phyllobacterium* and *Pannonibacter*, have been relatively under-researched, and their origins remain uncertain due to insufficient evidence. Moreover, their prevalence across studies was below 50% (present in fewer than six studies). In contrast, several genera commonly found in the gut and oral microbiota, including *Acinetobacter*, *Prevotella*, and *Clostridium sensu stricto-1*, were observed in more than half of the studies, and these genera not only showed higher abundance but also greater prevalence in the NCt group compared to the Ct group (Figure 3C-D). For the 20 genera with over 50% prevalence across studies and higher abundance in the NCt group, the overall trend indicated higher prevalence in NCt, though a few studies showed a reverse trend for individual genera (Figure 3D). These findings suggest that microbial signatures, particularly those derived from the gut and oral microbiota, are amplified in various clinical disorders. This amplification may be attributed to increased permeability of the barrier between organs and the bloodstream, driven by factors associated with disease pathologies, including microbial dysbiosis, inflammation, and immune dysregulation.^8^

## Discussion

### Blood microbiome research with amplicon sequencing

Recent studies on the blood microbiome have primarily employed either metagenomic shotgun sequencing or amplicon sequencing. Metagenomic shotgun sequencing is generally considered an advanced technique, offering enhanced detection of bacterial species with high accuracy and the ability to predict functional putative genes.^93^ Notably, a recent large-scale study using metagenomic shotgun sequencing concluded that there is no consistent core microbiome endogenous to human blood.^109^ Instead, it suggested that commensal microorganisms transiently and sporadically translocate from other body sites into the bloodstream, contradicting our findings. This discrepancy may be attributed to differences in sequencing techniques, as although amplicon sequencing has certain limitations compared to metagenomic shotgun sequencing – such as lower resolution, an inability to detect non-bacterial microorganisms (in the case of 16S amplicon sequencing), and primer-based biases – it remains a reliable method and could offer advantages over metagenomic approaches for blood microbiome research.

Blood typically contains a low microbial biomass but a high concentration of host DNA, primarily derived from the nuclei of leukocytes. The concentration of host DNA can vary based on the host’s immune status, tending to decrease during immunosuppression and increase during inflammation.^54,105^ Even in cell-free blood components like plasma, there is still a significant amount of non-bacterial DNA, including cfDNA, which is largely composed of mitochondrial DNA.^13^ This host DNA often outweighs microbial cfDNA, complicating metagenomic analyses. These characteristics – high host DNA content and low microbial biomass – pose significant challenges for metagenomic shotgun sequencing, as the overwhelming presence of non-microbial signals diminishes the sensitivity for detecting microbial signatures.^88,102^ Similar to blood, saliva, nasal cavity, and skin samples typically yield over 90% of human genome-aligned reads in metagenomic sequencing, necessitating the use of protocols that deplete host DNA prior to sequencing.^75^ However, one study^102^ reported that even after a 1000-fold reduction in host DNA from gastric biopsy samples, the ratio of microbial to host DNA still remained approximately 1:10^6^, highlighting the persistent challenges of host DNA contamination in low-microbial-biomass samples. An emerging approach to address these challenges is the 2bRAD-M method, which employs Type IIB restriction enzymes to generate iso-length DNA fragments that are sequenced.^106^ This method has shown promise in generating accurate species-level taxonomic profiles from samples with high host DNA contamination, low microbial biomass, or severely degraded DNA. In comparison, amplicon sequencing is relatively less affected by host DNA contamination compared to metagenomic shotgun sequencing, as it selectively amplifies microbial DNA using target-specific primers. However, it is not entirely free from this issue. Off-target amplification of host DNA can still occur in samples with a high host-to-microbial DNA ratio.^22^ While the degree of host DNA contamination in amplicon sequencing is generally less severe than in metagenomic shotgun sequencing, it remains a concern that can affect the detection of low-abundance microbial taxa. To address the issue of host DNA contamination in amplicon sequencing, emerging techniques like Peptide Nucleic Acid (PNA) clamping and blocking oligonucleotides are proving effective.^76,77,94^ These small molecules bind to mitochondrial and non-target host sequences during PCR amplification, preventing DNA polymerase from recognizing them, while selectively enhancing bacterial DNA amplification. Studies have demonstrated that PNA clamping significantly reduces host contamination, improving the accuracy of bacterial profiling in samples with high host-to-microbial DNA ratios. For example, PNA clamping has led to a 13-fold increase in bacterial sequences compared to traditional methods, facilitating more accurate microbial analysis by reducing the amplification of mitochondrial and chloroplast DNA.^113^ PCR enrichment helps detect low-abundance microbial taxa by enhancing their signals, though contamination from reagents and library preparation can introduce background noise, potentially diluting the reads of low-abundance microbes. Despite these challenges, with proper decontamination methods and sufficient sequencing depth, amplicon sequencing remains a viable and often advantageous method for profiling microbial communities in low-biomass samples.

### Common blood microbiome

The idea that microorganisms can exist in circulatory fluids, such as blood – which were previously considered sterile – is a recent area of research. Numerous studies have reported microbial DNA in the blood of healthy individuals, and some have demonstrated the presence of viable bacteria through culture-based methods, indicating that the microbial signatures in blood are not solely derived from cfDNA but also include actual living bacteria.^84^ Building on these findings, many efforts have been made to identify a “common blood microbiome,” similar to research conducted on the gut microbiome, which can be used to define a healthy status based on overall microbial diversity and composition. However, it is important to note that the bloodstream is not a habitat that requires microbes for symbiotic interaction with the host, unlike the gut, oral cavity, and vagina. Additionally, it is not an ideal environment for microbial growth, as it is constantly patrolled by immune cells such as neutrophils, macrophages, and lymphocytes, which rapidly detect and eliminate microbes.^95^ In this context, a certain amount of viable bacteria in the bloodstream is referred to as bacteremia, which generally does not cause illness. However, if the bacterial load exceeds a specific threshold that overwhelms the immune system’s ability to manage it, the condition is termed septicemia, which induces severe systemic inflammation, including a cytokine storm.^14^ Moreover, under normal circumstances, the high flow rates and shear forces present in the bloodstream challenge microbes to attach to surfaces and multiply.^111^ In light of these facts, defining a common blood microbiome should involve viewing it as a shared microbial signature rather than merely a collection of microbes present in circulation.

Microbial signatures in the bloodstream are generally believed to translocate primarily from microbiota-rich areas of the body or may be introduced during medical interventions, both physical and surgical. While there is a small chance, environmental microbes can also enter the bloodstream through injuries, insect bites, scratches, animal bites, or even during activities like brushing teeth.^122^ Interestingly, previous studies have compared blood microbiome data from healthy individuals with microbiome data from the Human Microbiome Project and found that the overall blood microbiome overlaps with skin and oral microbiomes, rather than the gut microbiome, which has the highest microbial load.^37,114^ This discrepancy may be attributed to contamination from skin and environmental microbiota during blood sample handling. Environmental microbiota, such as those from aerosols or surfaces, are closely linked to the oral and skin microbiomes.^119^ Thus, if contamination is not adequately controlled, blood microbiome data could be biased toward oral and skin microbes, regardless of actual microbial signatures from the gut. A more recent study, with rigorous quality control, found microbial signatures in blood primarily originating from the gut, oral cavity, and genitourinary tract, further supporting this assumption.^109^

Genera such as *Corynebacterium*, *Streptococcus*, and *Lactobacillus*, which have an overall prevalence of over 50% in healthy individuals, are commonly found throughout the human body, including the oral cavity, gastrointestinal tract, skin, and vagina. Among these, *Corynebacterium* shows the highest prevalence and is primarily innocuous, often forming commensal relationships with the host.^77^ However, some species can occasionally cause severe infections, such as sepsis or endocarditis, particularly in immunocompromised individuals. For example, *Corynebacterium jeikeium* is a clinically significant non-diphtheria species that can lead to various infections in patients with underlying risk factors and comorbidities.^10,11^ Additionally, genera with an overall prevalence exceeding 40%, such as *Bacteroides* and *Enterococcus*, are primarily found in the gut and play essential roles in maintaining intestinal health. During gut dysbiosis, *Bacteroides fragilis* can cause serious infections, such as peritonitis or sepsis, especially in immunocompromised individuals.^24,127^ Similarly, *Enterococcus* can act as an opportunistic pathogen, spreading to lymph nodes, blood, liver, and spleen when the immune system is weakened or the gut barrier is compromised.^25,58^ Dysbiosis of the oral microbiome and the translocation of periodontopathic microorganisms into the bloodstream can trigger both local and systemic inflammation. Activities such as chewing, tooth brushing, periodontal probing, scaling, and dental extractions can facilitate bacterial entry into capillaries, allowing these bacteria to enter systemic circulation. Pathogenic oral bacteria, such as *Porphyromonas gingivalis*, associated with inflammatory oral diseases, can translocate into the bloodstream and lead to systemic inflammation.^39,40^ Notably, the brain tissue of Alzheimer’s patients has been found to contain *P. gingivalis*, suggesting it may travel from the oral cavity to the brain via bloodstream.^27^

Taken together, blood microbiomes contain microbial DNA derived from commensals in microbiota-rich areas, primarily the gut and oral cavities, which can be considered a “common blood microbiome.” The overall composition of the blood microbiome can vary based on individual microbiome status and health, influencing the permeability of microbial signatures from various body sites into circulation. Although common microbial signatures consist mainly of genera that are largely innocuous commensals, some species are classified as opportunistic or harmful pathogens. Future studies employing sequencing strategies that ensure species-level resolution, such as full-length 16S amplicon sequencing, would provide more informative insights into health risks associated with the blood microbiome. Additionally, testing the viability of microbes is essential. For instance, Propidium monoazide (PMA) is a dye that selectively enters cells with damaged membranes, marking them as dead. Once inside, PMA binds to the DNA, preventing amplification during PCR, which allows analysis to focus specifically on bacteria with intact membranes, effectively distinguishing viable cells from dead ones.^80^ Thus, PMA treatment combined with sequencing could serve as a robust strategy for analyzing only viable microbes in blood. While this method has limitations for quantitatively assessing complex microbial communities,^120^ it remains a promising approach, especially given that blood is a relatively less complex microbial environment.

### Amplification of microbial signatures in clinical disorders and their association with the immune system

Numerous studies have reported changes in circulating microbial DNA across various disease states and medical procedures. Efforts to identify microbial signatures in blood as predictive or diagnostic markers are ongoing.

In patients with cardiovascular diseases (CVD), elevated levels of circulating cfDNA, particularly microbial DNA, have been observed, often exceeding levels of human cfDNA compared to healthy controls.^26^ Case-control studies have shown that patients with myocardial infarction (MI) and chronic coronary syndrome exhibit a reduction in blood microbiome diversity compared to healthy individuals.^2,55^ Notably, the genera *Kocuria* and *Enhydrobacter* were found to positively correlate with cardiovascular mortality.^64^ Additionally, a reduction in Bacteroidetes and an increase in *Bifidobacterium* have been linked to MI, suggesting these alterations could serve as biomarkers for cardiovascular disease.^48,56^ Additionally, *Streptococcus* has been associated with infective endocarditis and atherosclerosis, indicating its role in cardiovascular inflammation and plaque formation, while *Acinetobacter* species found in atherosclerotic plaques may contribute to endothelial dysfunction and increased cardiovascular risk.^5,99^ Beyond direct bloodstream infections, gut-derived bacteria may also influence CVD through systemic inflammation and metabolic dysregulation. *Romboutsia*, associated with gut dysbiosis, may exacerbate systemic inflammation, a key driver of CVD progression.^123^ These findings underscore the need for further research to explore the mechanistic pathways linking microbial taxa to CVD and their potential clinical applications.

In type 2 diabetes mellitus (T2DM), patients also exhibit altered blood microbial profiles compared to controls. A large-cohort longitudinal study identified a significant association between higher baseline circulating bacterial DNA and the onset of T2DM.^3,99^ Notably, *Staphylococcus* species have been found in higher abundance in diabetic patients, where they may contribute to systemic inflammation and insulin resistance.^104^ Similarly, *Acinetobacter*, commonly associated with nosocomial infections, has been detected in diabetic foot ulcers and is linked to poor wound healing and persistent inflammation, exacerbating complications in diabetic patients.^88^

Liver diseases, such as liver cirrhosis, have been extensively studied for their close association with the gut microbiome. Microbial translocation from the gut to the liver through portal circulation has been proposed as a key mechanism in the progression of liver disease. Consequently, patients with liver conditions may exhibit higher microbial loads and PAMPs in their blood, especially in the context of gut dysbiosis.^41,103,117^ Among the bacterial genera frequently detected in the blood of liver disease patients, *Acinetobacter* have emerged as potential biomarkers. *Acinetobacter* species, known for their endotoxin production and association with hospital-acquired infections, have been detected at higher levels in cirrhotic patients. Their presence in the bloodstream may exacerbate hepatic inflammation by triggering innate immune responses, leading to further liver damage and an increased risk of complications such as hepatic encephalopathy.^45^

In cancer, distinct blood microbiome profiles have been observed across various types. For instance, breast cancer patients were found to have elevated levels of *Citrobacter*, *Bacteroides*, *Enterobacter*, and *Bifidobacterium*, while *Staphylococcus*, *Lactobacillus*, *Fusobacterium*, *Porphyromonas*, and *Actinomyces* were depleted.^4^ In advanced colorectal cancer, increased microbial diversity was observed in patients responding to immunochemotherapy, with elevated baseline levels of gut-origin bacteria such as *Bifidobacterium* and *Enterococcus* and reduced *Pseudomonas*.^124^

Recent findings suggest that microbial translocation into the bloodstream may contribute to neuroinflammation in Alzheimer’s disease (AD). Pathogenic oral bacteria, such as *Porphyromonas gingivalis*, have been identified in the brain tissue of AD patients, indicating that microbes may travel from peripheral sites to the brain via circulation.^27^ Furthermore, AD patients’ brains contain higher bacterial loads compared to controls, implying increased circulating microbiome and microbial DNA entering the brain.^1,30^ Additionally, AD patients showed higher levels of *Romboutsia* than cognitively normal controls.^116^ While the role of *Romboutsia* in neurodegenerative diseases is not well understood, a study in a rat model of type 1 diabetes with cognitive decline found that *Romboutsia* negatively correlated with hippocampal lactate and myo-inositol levels but positively correlated with serum glutamine and hippocampal GABA levels. This suggests that *Romboutsia* may influence cognitive function by modulating the glutamate-glutamine (GABA) cycle, a key pathway involved in neurotransmission.^34^ These findings indicate a potential link between *Romboutsia* and neuroinflammation in AD, warranting further investigation into its role in disease progression.

Overall, these findings indicate significant alterations in blood microbiome profiles across various diseases, potentially influencing disease progression. This also suggests that specific circulating microbial profiles may hold prognostic value in different clinical conditions and highlights the potential of blood microbiota as noninvasive biomarkers for monitoring treatment efficacy. To achieve this, it is essential to understand the origin of these microbes and microbial DNA, as well as the mechanisms driving their translocation in clinical disorders.

**Figure 4.**
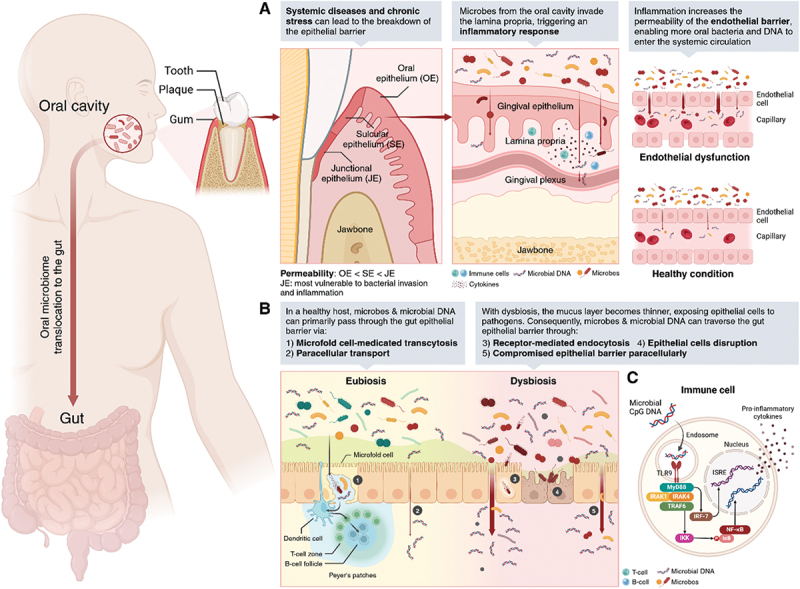
Trajectory of microbes and microbial DNA translocation from oral cavity and gut to circulation. (A) Oral translocation: systemic diseases and chronic stress can compromise the epithelial barrier of the gums, increasing the risk of bacterial invasion from the oral environment, including dental plaque–a microbial biofilm with a high microbial load. The junctional epithelium, which is attached to the tooth, is particularly vulnerable and more permeable compared to sulcular and oral epithelium. Microbes from the oral cavity can infiltrate the connective tissue (lamina propria), triggering an inflammatory response and cytokine production. Consequently, inflammation disrupts the endothelial barrier of the gingival plexus, allowing oral microbes and microbial DNA to enter systemic circulation. (B) Gut translocation: In healthy individuals, microbes or microbial DNA can still translocate into the connection tissue via M-cell-mediated transcytosis or paracellular transport through gaps between epithelial cells. However, during dysbiosis–often observed in patients with clinical disorders–the mucus layer protecting gut epithelial cells thins, exposing the epithelium to pathogens. Some pathogens may exploit receptor-mediated endocytosis or disrupt epithelial cells using endotoxins. Furthermore, as the gaps in the epithelial barrier widen, more microbes and microbial DNA can pass through the gut tissue, eventually entering systemic circulation. (C) Inflammatory response to microbial DNA: microbial DNA, which contains CpG motifs, is recognized as a pathogen-associated molecular pattern (PAMP) by Toll-like receptor 9 (TLR9). TLR9 activation triggers a signaling cascade involving MyD88, which associates with IRAK1 and IRAK4. This leads to the activation of TRAF6, which in turn activates the IκB kinase (IKK) complex. Phosphorylation of IκB proteins by IKK results in their degradation, allowing nf-κB to translocate to the nucleus, where it binds specific DNA sequences to promote the transcription of pro-inflammatory cytokines. Additionally, MyD88 activates IRF-7, which binds to interferon-stimulated response elements (ISREs) to drive the transcription of type I interferons and other antiviral genes. Created in BioRender (bioRender.com/q44u940).

Given the microbial loads across the body, the gut and oral cavity are primary regions harboring a high concentration of microbes, making them critical sites for potential translocation of microbial DNA into circulation. In various clinical disorders, dysfunction of the epithelial barrier has been frequently observed. Among 12 clinical studies included in a meta-analysis, three assessed microbial translocation by measuring intestinal permeability markers or comparing blood microbiome profiles with those of the gut and oral cavity.^35,125,127^ These studies reported elevated levels of fatty acid-binding protein 2 (FABP2), lipopolysaccharides (LPS), and D-lactate in circulation in disease cohorts compared to healthy controls.^35,127^ Additionally, blood microbiome composition showed notable similarity to the oral (18%) and gut (21%) microbiomes,^125^ suggesting compromised barrier integrity during disease progression. This disruption may act as a trigger for clinical disorders by provoking immune responses or may arise as a consequence of disease.^85,99^ Notably, poor oral health and periodontal disease are closely linked to systemic conditions, with oral microbiome alterations both contributing to and resulting from disease, underscoring their interconnected nature.^36,68,77^ When the epithelial barrier of the gums is compromised, the risk of bacterial invasion from the oral environment, particularly from dental plaque – a microbial biofilm with a high microbial load – significantly increases.^78^ The junctional epithelium, which attaches to the tooth, is especially vulnerable and more permeable compared to the sulcular and oral epithelia.^110^ This allows microbes from the oral cavity to infiltrate the connective tissue (lamina propria), triggering an inflammatory response and cytokine production. As inflammation disrupts the endothelial barrier of the gingival plexus, oral microbes and microbial DNA can enter systemic circulation.^43^ This suggests that, in addition to the low levels of microbial DNA that translocate to the circulation under healthy conditions, individuals with gingivitis, periodontitis, or systemic inflammation – who are more prone to endothelial permeability disruption – may show increased microbial signatures in their blood microbiome (Figure 4A). Similar mechanisms are observed in the gut epithelium. Even in healthy individuals, microbes or microbial DNA can translocate into the connective tissue via microfold cell (M-cell)-mediated transcytosis or through paracellular transport between epithelial cells.^49,62^ M-cells have a unique ability to uptake antigens from the small intestine lumen via endocytosis, phagocytosis, or transcytosis, delivering them to antigen-presenting cells, such as dendritic cells and B lymphocytes, within Peyer’s patches.^59^ However, various pathogens exploit M-cells to cross the gut epithelial membrane and can even induce transcytosis of other commensal bacteria.^89,50,17,18^ In conditions of dysbiosis, commonly observed in patients with clinical disorders, the protective mucus layer thins, exposing the underlying epithelium to pathogens. These pathogens may also utilize receptor-mediated endocytosis^19^ or disrupt epithelial cells through endotoxins.^73^ As gaps in the epithelial barrier widen, more microbes and microbial DNA can penetrate the gut tissue, eventually entering systemic circulation (Figure 4B). While translocated microbes can clearly induce an immune response, microbial DNA also triggers inflammatory cascades. Microbial DNA contains CpG motifs, which are recognized as PAMPs by TLR9.^6^ TLR9 activation initiates a signaling cascade involving MyD88, which interacts with IRAK1 and IRAK4, leading to the activation of TRAF6. TRAF6 then activates the IκB kinase (IKK) complex, which phosphorylates IκB proteins, marking them for degradation. This allows NF-κB to translocate to the nucleus and bind to specific DNA sequences, promoting the transcription of pro-inflammatory cytokines.^108^ Additionally, MyD88 activates IRF-7, which binds to interferon-stimulated response elements (ISREs), driving the transcription of type I interferons and other antiviral genes^44,53^ (Figure 4C).

Taken together, the increased microbial signatures observed in patients with clinical disorders may be indicative of chronic inflammation and a compromised epithelial barrier, enabling greater translocation of microbial signals into circulation. As circulating microbes and microbial DNA further exacerbate inflammation, abnormalities in the blood microbiome – regardless of microbial viability – should be considered potential clinical signs when compared to control groups.

### Limitations of research on blood microbiome

In this study, a significant proportion of microbes detected in the blood could not be identified using the Disbiome database (Figure 2A,B). This raises important questions regarding the nature of the blood microbiome and its complexity. First, the presence of microbes in the bloodstream is often transient or reflects microbial cfDNA from other body sites, such as the gut, oral cavity, or skin. These microbial signals may not be from active colonizers, but rather from bacteria or their fragments that have translocated into the bloodstream through mechanisms such as compromised barrier function.^71^ Given this, microbes detected in blood may not have clear matches in the Disbiome database, which primarily catalogs microbes based on more traditional body sites like the gut or oral cavity.^47^ Second, the low microbial biomass in blood presents challenges in profiling microbial communities accurately.^28^ Since the microbial diversity of blood has not been thoroughly explored in comparison to other body sites, many microbes present in blood may not yet be represented in these databases, explaining the high proportion of unidentified taxonomy.^12^ Disbiome database is primarily constructed from data obtained from environments with high microbial biomass, such as the gut and oral cavity. These databases may not adequately cover microbes found in low-biomass environments like blood. Third, although quality control measures were implemented to minimize contamination, the possibility of external microbial contamination cannot be entirely ruled out. Microbes introduced during sample processing, such as from DNA extraction kits or reagents, may be misidentified as genuine blood microbes.^28^ These contaminant sequences might not be found in databases, leading to a higher number of unidentified microbes. The fact that a substantial portion of blood microbes remain unidentified in the Disbiome database underscores the complexity and the largely unexplored nature of the blood microbiome. These results suggest that further research and expanded microbial databases are necessary to fully understand the diversity of microbes in the bloodstream, especially in relation to health and disease.

## Conclusion

In this study, through a meta-analysis of publicly available amplicon sequencing data, we demonstrated that amplicon sequencing remains a feasible and reliable strategy for blood microbiome research, provided sufficient sequencing depth is achieved. This approach becomes even more beneficial when using amplicon locations and lengths that ensure species-level resolution, alongside up-to-date strategies to eliminate potential environmental contaminants during sample handling and reagent preparation. The presence of microbial DNA in the bloodstream, primarily originating from the gut and oral microbiota, forms a “common blood microbiome,” with its frequency and abundance varying based on individual microbiome profiles and health status, reflecting the permeability of microbial signals from different body sites into circulation. In individuals with clinical disorders, elevated microbial signatures may indicate chronic inflammation and compromised epithelial barriers, facilitating increased microbial translocation. Regardless of microbial viability, changes in the blood microbiome can further exacerbate inflammation, making these abnormalities valuable potential biomarkers for clinical diagnosis when compared to healthy controls. Despite the identification of common microbial signatures, the considerable variation in microbial frequency and abundance across studies highlights the importance of including control groups with similar demographics for accurate comparative analysis with case groups. Additionally, to more accurately assess the blood microbiome, it is essential to include negative controls at each experimental step, including sample collection, blood preprocessing, amplification, and sequencing. Utilizing standardized methodologies, such as those proposed by the Earth Microbiome Project, will be crucial for future studies.

## Supplementary Material

Supplementary material.pdf

## Data Availability

The data that support the findings of this study are openly available in NCBI-SRA at https://www.ncbi.nlm.nih.gov/sra., with the reference number provided in Table 1.
