# Supplementary material for: Common microbial signatures in blood and their amplification in clinical disorders
Source: Gut Microbes Rep. 2025 Mar 19;2(1):2473450. doi: 10.1080/29933935.2025.2473450 (PMC12940139; doi:10.1080/29933935.2025.2473450)
Supplement: Supplementary material.pdf [file KGMR_A_2473450_SM2770.pdf]

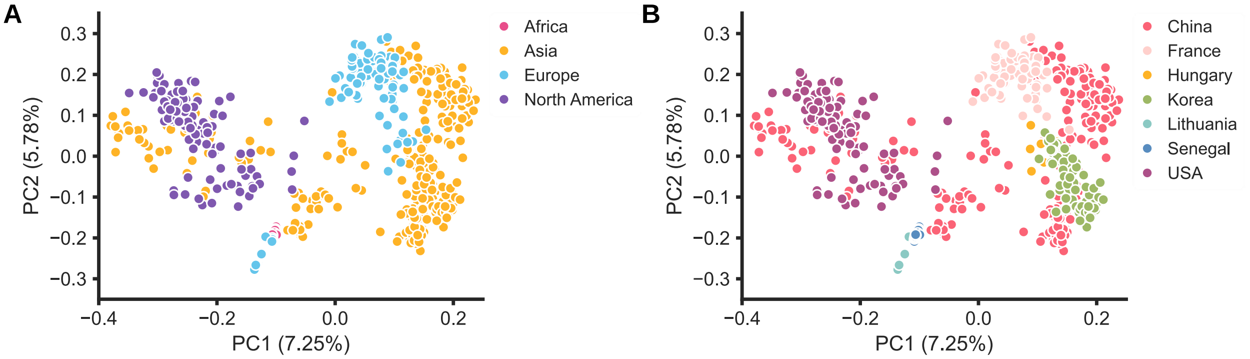

Figure S1. Study Characteristics. PCoA plots showing sample distribution based on unweighted UniFrac distances, grouped by (A) continent and (B) country.
